# The Loop-Mediated Isothermal Amplification Technique in Periodontal Diagnostics: A Systematic Review

**DOI:** 10.3390/jcm10061189

**Published:** 2021-03-12

**Authors:** Marcin Lenkowski, Kacper Nijakowski, Mariusz Kaczmarek, Anna Surdacka

**Affiliations:** 1 Department of Conservative Dentistry and Endodontics, Poznan University of Medical Sciences, 60-812 Poznan, Poland; annasurd@ump.edu.pl; 2 Department of Cancer Immunology, Poznan University of Medical Sciences, 61-866 Poznan, Poland; markacz@ump.edu.pl

**Keywords:** loop-mediated isothermal amplification, periodontal disease, periodontal pathogen, periodontal bacteria, periodontal diagnostics

## Abstract

The course of periodontal disease is affected by many factors; however, the most significant are the dysbiotic microflora, showing different pathogenicity levels. Rapid colonization in the subgingival environment can radically change the clinical state of the periodontium. This systematic review aims to present an innovative technique of loop-mediated isothermal amplification for rapid panel identification of bacteria in periodontal diseases. The decisive advantage of the loop-mediated isothermal amplification (LAMP) technique in relation to molecular methods based on the identification of nucleic acids (such as polymerase chain reaction (PCR or qPCR) is the ability to determine more pathogens simultaneously, as well as with higher sensitivity. In comparison with classical microbiological seeding techniques, the use of the LAMP method shortens a few days waiting time to a few minutes, reducing the time necessary to identify the species and determine the number of microorganisms. The LAMP technology requires only a small hardware base; hence it is possible to use it in outpatient settings. The developed technique provides the possibility of almost immediate assessment of periodontal status and, above all, risk assessment of complications during the treatment (uncontrolled spread of inflammation), which can certainly be of key importance in clinical work.

## 1. Introduction

Periodontitis is a chronic inflammatory state leading to tooth-supporting apparatus break-down, which as a consequence could lead to partial or total tooth loss. Dysbiotic microbes along with environmental, genetic and host factors are the main components influencing the initiation and progression of this disease [1]. Several systemic conditions such as diabetes mellitus, stress, obesity and acquired immunodeficiency syndrome cause an exacerbated inflammatory state promoting the development of periodontal inflammation. An excessive level of glucose, as well as the accumulation of advanced glycation end-products (AGEs), cause a pro-inflammatory cascade [2]. Smoking leads to significant microvascular constriction, masking the clinical signs of bleeding on probing [3]. Furthermore, pharmacological agents can lead to gingival enlargement resulting in pseudo pocketing. Vitamin C deficiency predisposes to an increased propensity for bleeding [4]. Although occlusal trauma was once considered a predisposing factor, it has only been observed in an animal model, and there is a lack of evidence for such a phenomenon in humans [5].

The clinical diagnosis of periodontal disease is substantially dependent on parameters such as clinical attachment loss, pocket probing depth, bleeding on probing, plaque index, mobility, furcation involvement, and radiographic evaluation of bone architecture. These indicators, however, do not display the current state of the disease and do not yield information about the activity or risk for disease advancement [6,7].

Clinical diagnosis has its limitations and does not enable clinicians to determine the cause, pathogenesis or prognosis in case of an advanced stage of the periodontal disease. Therefore, it is thought that the utilization of available technology such as molecular analysis could aid in the determination of the qualitative and quantitative composition of periodontal pathogens. An exact determination of the composition of periodontopathic microflora could be instrumental in the process of development of a detailed, accurate therapeutic regimen [8].

In a healthy periodontal state, the count of intraoral bacteria averages around 1 × 10^9^, whereas in case of periodontal support break-down, these amounts exceed counts of 1 × 10^87^ [9]. Evaluation of subgingival biofilm composition by microbiological and molecular methods has revealed an association with a large number of microbes, some of which are capable of periodontal tissue destruction [10,11]. In 1998, Socransky and Hafajee studied the behavior of bacteria and grouped them into several subclasses based on their pathogenicity and colonization abilities within the subgingival environment [12]. They postulated, that the initial colonizers, although usually non-pathogenic, belonging to yellow, blue, green and purple complexes, act as the initiators of biofilm formation [13]. However, it has also been discovered that these species provide adhesive properties to microbes from an orange complex. In turn, this could lead to ideal conditions for species such as *Porphyromonas gingivalis*, *Treponema denticola* and *Tannerella forsythia*, part of the red complex, known as a main culprit in chronic periodontal disease, causing increased pocket probing and spontaneous bleeding [14]. Although *“*red complex*”* bacteria predominate within periodontal pockets, another pathogen, *A. actinomycetemcomitans*, a Gram-negative bacterium, especially serotypes A, B and C, plays a significant role in the rapid progression of periodontal disease [15]. Amongst many other bacteria implicated in the progression and development of periodontitis are *Prevotella intermedia*, *Campylobacter rectus*, *Peptostreptococcus micros*, and *Spirochetes species* [16,17]. It was indicated also that various viral agents, such as herpes viruses, are found to be actively involved in the process of aggressive periodontitis [18]. Additionally, a plethora of various fungal agents including *Candida albicans* have been isolated in individuals with primary and acquired immunodeficiencies, playing a role in the destructive process of periodontal support in collaboration with other periodontopathogens [19,20].

Culturing methods were once coined as a golden standard. As with any procedures, traditional microbiological methods have their advantages, but also several limitations. Most of the pathogens present in deep periodontal pockets are anaerobic and require specific growth conditions, implying issues with cultivation, sampling and transportation, potentially resulting in erroneous diagnostic outcome. Difficulties also include bacterial vitality maintenance, extensive waiting periods before diagnosis, inability to discriminate between closely related taxa, and low detection values of 10^3^–10^4^ bacterial cells. Cultivation methods are capable of identifying oral pathogens, although they fail to do so for a known putative periodontopathogen such as *T. forsythia* [21,22].

A growing need for precision, rapid detection, and quantification of periodontal pathogens required other methods to be designed. Techniques such as flow cytometry, DNA–DNA hybridization, immunochemical assays, or enzymatic methods have been utilized. Unfortunately, their questionable specificity and sensitivity deemed these tests unreliable [23,24].

As molecular techniques emerged, polymerase chain reaction (PCR) was developed as a diagnostic tool for a more accurate, sensitive and rapid assay for multiple periodontal pathogens such as *A. actinomycetemcomitans*, *P. gingivalis*, *P. intermedia*, *T. forsythensis* and *T. denticola* [25,26].

Although PCR is a sensitive method for the detection of microbes, is it prone to errors due to the presence of DNA polymerase inhibitors, present in clinical samples. Hemoglobin, heparin and EDTA (ethylenediaminetetraacetic acid) present during sample acquisition, and alcohols, detergents and salts present throughout the DNA isolation process, can lower the efficiency of the reaction and even inhibit it. Also, excessive de-thawing can significantly alter its diagnostic potential. Another limitation is the need for expensive specialized equipment, which besides its high cost, is disqualified from usage outside of well-controlled laboratory conditions [27,28].

For many years the CR technique has undergone many modifications, enabling expansion of its capabilities. Some of these adjustments include RT-PCR (reverse transcription-PCR)—a technique combining reverse transcription of RNA fragment; and PCR-RFLP (PCR-Restriction Fragment Length Polymorphism)—PCR reaction combined with restriction analysis of the amplification products.

The advent of this powerful assay has led to a more accurate tool for identification of total pathogen count through the development of species-specific primers, which do not amplify non-target sequences. The qPCR with species-specific primers provides accurate quantification of individual microbial species and total bacterial count in dental plaque samples. This method has been deemed a gold standard for the identification of etiological factors involved in the progression of periodontal diseases [29,30,31].

As technology became more advanced, a new method of combining reverse transcription with qPCR, qRT-PCR, has been designed. Besides its capability in detecting anaerobic oral bacteria, it enabled detection of DNA of viable and non-viable cells, providing more information pertaining to the activities and current states of microbiota, and by doing so providing very accurate feedback as to the current status of the disease [32].

The loop-mediated isothermal amplification (LAMP) technique has been developed by Notomi et al. [33]. This novel molecular tool has been utilized for bacterial identification due to its superior specificity, efficiency, and ease of management. Lately, various commercial LAMP kits have been introduced to the market, including tests for detection of *Salmonella*, *Escherichia coli*, and *Listeria monocytogenes* [34]. Identification through LAMP was also performed for DNA viruses such as HSV, Adenovirus, HBV, HSV-1, HSV-2, and VZV-1. RNA viruses were detected through the incorporation of a reverse transcriptase enzyme to detect the envelope capsid protein of a West Nile virus [35,36,37]. Additionally, this technique has been employed for parasite detection, e.g., *Toxoplasma* [38]. Interestingly, it has also been developed for bovine sex type identification using ethidium bromide and CuSO_4_, and detection of genetically modified food by combining LAMP with immunochromatography [39].

Modern periodontology is accelerating at a spectacular speed in the scientific and technological areas concerning diagnosis (e.g., in-office molecular tests [40,41]) and treatment (e.g., use of stem cells with nanomaterials for bone regeneration [42,43]).

The present systematic review has been designed to answer the question “Is the loop-mediated isothermal amplification technique useful for diagnosis of periodontal diseases?”, formulated according to PICO (“Population”, “Intervention”, “Comparison”, “Outcome”).

## 2. Results

**Figure 1:**
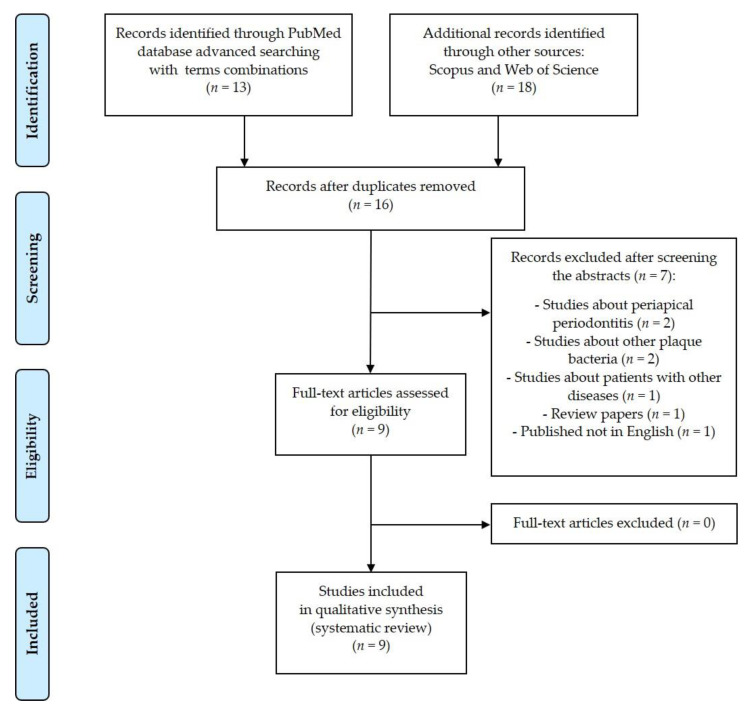
PRISMA flow diagram presenting the detailed search strategy.

In this systematic review, nine studies following the search criteria were included. Figure 1 shows the detailed selection strategy of the articles. The inclusion and exclusion criteria are presented in section Materials and Methods.

From each study included in the present review, data on its general characteristics such as year of publication and setting, participants involved, methods of sample collection and storing, and detected periodontal bacteria, are reported in Table 1. Table 2 presents LAMP parameters such as the composition of the reaction mixture, reaction conditions and methods for the detection of products. Additionally, the evaluation of LAMP sensitivity and specificity for particular pathogens is discussed in Section 3.2.

The presented results confirm the usefulness of the LAMP technique for the diagnosis of selected perio-pathogens.

## 3. Discussion

### 3.1. Loop-Mediated Isothermal Amplification Method—Principles and Limitations

There is clearly an expanding demand for new methods not requiring advanced equipment. LAMP is considered a potent tool due to its advantageous properties such as the elimination of the DNA denaturation step, DNA polymerase exhibiting displacement ability, high specificity due to four primers and enhancement of efficiency due to isothermal conditions by eliminating time loss [33,53]. Other advantages include minimal investment in a conventional heating block or a water bath necessary to acquire isothermal conditions under which the LAMP reaction takes place. Its ability to rapidly detect bacteria without significant influence of ever-present non-specific DNA sequences also makes it an ideal solution [54,55]. LAMP, as well as other isothermal (60–70 °C) techniques, eliminates the need for the DNA denaturation step through the use of GspSSD polymerase, due to its capacity for strand displacement. It consists of a set of at least four specific primers, including two outer primers F3 and B3, and two inner primers FIP and BIP along with loop primers. A stem-loop DNA is constructed from sequences of DNA derived from the internal primer structure. A 3′-prime terminus of the stem-loop is the initiation site for the DNA synthesis. Subsequently, one inner primer binds the loop to the LAMP product ensuring a strand displacement activity and in effect producing the original stem-loop DNA along with a new stem-loop DNA twice the length of the original loop. The final products are stem-loops of the original target DNA sequence generated in about an hour. It also allows for very efficient and specific amplification of a given DNA fragment due to the utilization of at least two complementary DNA primers, but if it necessary, up to six primers could be used, providing extremely high specificity, due to hybridization to 100–250 basis pair (bp), allowing for quick pathogen identification: 10^9^ copies created in less than 30 min. Real-time monitoring and establishment of a number of DNA copies in a closed tube system minimizes the risk of contamination. Furthermore, if necessary, the final result of the reaction can be observed with the naked eye with an addition of a fluorescent dye [53,54,55].

The majority of the above-mentioned methods can be performed in ideal laboratory conditions with the use of sophisticated equipment under an extended working time. However, the LAMP technique has utilized many methods for detection of its end-products. One of the methods detects the turbidity achieved through the usage of magnesium pyrophosphate, Mg_2_P_2_O_7_, as an end-product, at an optical density of 650. This method has been used in the real-time monitoring process for the presence of microorganisms [56]. The drawbacks of this technique are the long incubation time (≥60 min), and the reception product can be hard to visualize even under ideal conditions [57].

Other methods for direct detection of end-products utilize intercalating, calcein, SYBR green I and HNB. Its addition prior to isothermal incubation creates an insoluble complex, making a fluorescent manganese–pyrophosphate reaction. A positive reaction leads to the formation of an orange color, whereas a negative end-result remains dark [58]. The use of SYBR green I dye is another alternative. Although this intercalating agent has reported good sensitivity, its drawbacks include LAMP inhibition by aerosol contamination. An issue caused by aerosol contamination has been solved by the use of HNB (Hydroxy-naphthol blue), yielding a blue final LAMP reaction. Unfortunately, this substance does not exhibit a fluorescent activity; therefore, only color observation with the naked eye is feasible [59].

Other drawbacks for calcein and hydroxy-naphthol blue include difficulty in color change perception. Furthermore, calcein requires incorporation of the ionic form of manganese, which can have an inhibitory effect on the polymerase reactions [60]. A further negative aspect of these particular dyes is the prolonged reaction time and relatively low sensitivity values of >100–1000 copies of DNA target [61,62]. Recently, however, another method which incorporates pH-sensitive dyes has been successful at eliminating the above-mentioned drawbacks.

Throughout the process of deoxy-nucleoside triphosphate incorporation into the new DNA strand, a DNA polymerase releases two by-products: a pyrophosphate moiety and a hydrogen ion. The proton release during this process has been utilized as the basis of various detection methods. An ability of the DNA polymerase to carry out its function within a range of a 2–3 pH unit drop allowed for the rapid detection of LAMP amplification without loss of effectiveness. This pH transition of an indicator dye is adequate for visualization of the final amplification product [63,64,65].

Recently, a new two-color RT LAMP assay was developed for detecting SARS-CoV-2 viral RNA, utilizing a set of specific primers for the N gene. When comparing this assay to the RT-qPCR, the detection rate of SARS-CoV-2 RNA was within the threshold (CT) of up to 30, with a sensitivity and specificity of 97.5% and 99.7%, respectively. A swab assay which did not require an RNA isolation step has also been developed. An assay sustained a high specificity value of (99.5%) but exhibited a slightly diminished sensitivity value of 86% compared to the original RT-LAMP assay [66].

Unfortunately, like any other techniques, the LAMP has several limitations. Since the final DNA product is composed of many large size concatemers and loop structures, this impedes its usage in the process of cloning [67]. A challenge, behind LAMPs high sensitivity and specificity, is that this is attained via the use of multiple (4–6) primers. A difficulty lies in the ability to obtain a correct target sequence for amplification for the relatively highly conserved region, specifically for a given microbe at 6–8 regions. The utilization of such a high number of primers also increases the risk of a false positive outcome due to primer–primer affinity, requiring further validation [33]. Since the stability of the LAMP product is very high and very difficult to degrade, it may increase the chances of contamination and, in effect, false-positive outcomes. Therefore, prevention of such a scenario is achieved through the use of filtered tips, specifically designed pipettes and a hood with a laminar flow of air. Moreover, the subjective nature of the turbidimetric and colorimetric LAMP assay makes the visual determination of the LAMP reaction somewhat challenging and relies on one’s perception of color [68]. Additionally, the ladder pattern of the final product, rather than a specific band pattern observed in the classic PCR reaction, makes product size determination impossible [67].

### 3.2. Loop-Mediated Isothermal Amplification Method in the Detection of Periopathogens

Despite these limitations, the LAMP technology could be utilized for the detection of periodontal pathogens. In order to test the sensitivity level of periodontopathic species, one of the research teams has utilized serial chromosomal serial dilutions to test the lower limit of the assay’s sensitivity. Yoshida et al. [45] established that, during one hour reaction time, the *P. gingivalis* primer set, without the loop primer set, had a limit of detection of 1 µg/tube. However, when loop primers were added, a faster detection rate has been demonstrated. For a 30-min reaction rate, the sensitivity was set at 1 μg/tube for chromosomal DNA. Moreover, the detection limits for the *T. forsythia* primer set were 10 fg/tube in a 40-min reaction without the loop primers and 10 fg/tube in a 20-min reaction with the loop primers, and for the *T. denticola* primer set were 100 ng/tube and 10 µg/tube, respectively. The added loop primers significantly improved the detection sensitivities for each bacterium. The amplification specificity was evaluated by restriction endonuclease digestion with NcoI (for the *P. gingivalis* amplicon), SnaBI (for the *T. forsythia* amplicon) and AluI (for the *T. denticola* amplicon).

On the other hand, the research group of Maeda et al. [44], achieved quantification of *P. gingivalis* by real-time monitoring of the LAMP reaction using SYBR Green I with linearity in the range of 10^2^–10^6^ cells, showing nearly identical results to conventional real-time PCR with the advantage of speed in favor of the LAMP method. They demonstrated high efficacy and specificity for the LAMP, which could be suitable for rapid oral bacteria screening and chairside diagnosis. In the study of Hamzan et al. [51], the detection limits of LAMP for *P. gingivalis* and *A. actinomycetemcomitans* were 10-fold more sensitive than the conventional PCR (for both perio-pathogens, at 1 ng and 10 ng, respectively). In a crude template of subgingival plaque, *P. gingivalis* and *A. actinomycetemcomitans* were detected by LAMP in 80% and 60% of tested samples, respectively, whereas using PCR, *P. gingivalis* was positive in 40% cases, and there is no significant detection rate for *A. actinomycetemcomitans*.

In contrast, Osawa et al. [46] designed LAMP primers which successfully amplified serotypes a–e of *A. actinomycetemcomitans* but no other periodontal bacteria. The amplification specificity was assessed by restriction endonuclease digestion with Sau3AI for the *A. actinomycetemcomitans* amplicon. The detection limits for the real-time turbidimetry were 5.8 *×* 10^2^–5.8 *×* 10^7^ copies per reaction tube. For the rapid LAMP detection of *A. actinomycetemcomitans* in clinical specimens, the results were analogous to conventional PCR. Furthermore, Seki et al. [48] suggested that only PCR techniques concurrently detected non-JP2 types of *A. actinomycetemcomitans*. However, the presence of these strains seems not to influence LAMP detection of the JP2 clone, associated with the progression of aggressive periodontitis in adolescents of North and West African descent.

Similarly, Elamin et al. [49] examined the presence of *A. actinomycetemcomitans* in Sudanese adolescents with aggressive periodontitis. The prevalence of JP2 clone and non-JP2 genotypes were evaluated using LAMP and PCR. Non-JP2 types were detected in 70.6% of periodontitis patients, but the JP2 clone was not determined in either the cases or the controls. Both identification methods showed identical results. This allows speculation that the precise identity of the etiological perio-pathogens can be confused by population differences with regard to ethnic, environmental and genetic factors. Additionally, in 2017, the same authors [50] reported the highest risks of aggressive periodontitis in a case of co-infection with *A. actinomycetemcomitans* and human cytomegalovirus or Epstein-Barr virus type 1 (odds ratios 39.1 and 49.0, respectively).

Another study by Miyagawa et al. [47] investigated the presence of eight different pathogens using the LAMP method. The 16s gene was used for the study, using six separate DNA sequences, although it is important to note the fact that this gene tends to be non-specific. LAMP products were detected by 2% agarose gel electrophoresis. DNA amplification was observed in all LAMP reactions for each periodontal pathogen with the extracted DNA template containing 10^3^ cells of all tested species. In contrast, combining the DNA of all seven templates of the individual strains (10^3^ cells of each species) showed no amplicon. The sensitivity of the 30-min LAMP declined compared with the 60-min LAMP. For the 30-min reaction, 100 template cells were required to detect *E. corrodens*, and ten cells in the case of the other seven perio-pathogens. LAMP only for *P. gingivalis*, *A. actinomycetemcomitans* and *P. intermedia* was applied to the clinical plaque samples but with sensitivity equal or higher than real-time PCR. The practicability for other periodontal bacteria should be elucidated.

The development of a colorimetric method for the detection of *Porphyromonas gingivalis*, *Tannerella forsythia* and *Treponema denticola* has been designed by Al-Hamdoni et al. [69]. They have used the classical phenotypic method and the LAMP colorimetric method using four pairs of primers targeting the 16SrRNA genes along with loop primers with Colorimetric Master Mix containing Bst DNA polymerase and phenol red to detect amplicon formation. The phenotypic examination showed the diversity of the characteristics of isolates of the same strain. In just 30 min, LAMP made it possible to identify individual strains of perio-pathogens from both extracted DNA and directly from whole cells, in a highly specific and rapid manner, through visual interpretation of the results.

A new isothermal detection method called MB-LAMP (molecular isothermal loop amplification) that combines the advantages of LAMP and qPCR was developed by Liu et al. [70]. The advantage of this assay was the application of a molecular warning probe, LFP (Loop Forward Probe) or LBP (Loop Backward Probe), minimizing non-specific amplification of DNA fragments, enabling a higher specificity than the traditional LAMP method. As reported by Su et al. [52], this offers an increased detection potential of *P. gingivalis* by targeting a specific fragment of the *P. gingivalis* genome as well as obtaining high sensitivity. Within 20 min, the limit of detection was only 10^−4^ pM or 10^−7^ ng/μL. An important aspect of this method was the fact that the extension of the reaction time did not allow the detection of lower concentrations of nucleic acids. Like the QPCR reaction, which detected the lowest pure plasmid concentrations at 38 cycles, positive results were generally considered to be false positives when the reaction time reached above 35 cycles. The method showed no cross-reaction with 14 other pathogens. The test also showed high diagnostic sensitivity (100%) and specificity (100%) compared to quantitative real-time polymerase chain reaction (real-time qPCR). MB-LAMP turned out to be a much faster method than real-time qPCR, which allowed for a more efficient diagnosis of the presence of a given pathogen. The average reaction time of MB-LAMP and qPCR was 14.16 and 26.69 min, respectively.

### 3.3. Loop-Mediated Isothermal Amplification Method—Future Research Directions

Additionally, as the majority of diagnostic tests require each pathogen to be tested individually, it would be of great value to design a multiplex LAMP assay which would create a viable solution to detect multiple pathogens within the same test-tube. This would significantly reduce the amount of time required for pathogen identification, consequently leading to a more precise differential diagnosis and faster treatment implementation. The multiplex LAMP has been developed for the detection of a Dengue virus. Four specific primer sets targeting 30 noncoding regions were placed within a single test tube. A colorimetric reaction utilizing an intercalating HNB dye was used, allowing for naked eye visualization. No cross-reactivity has been reported [71]. In 2017, Stratakos et al. [72] developed a multiplex LAMP for the detection of pathogenic and non-pathogenic *E. coli* strains, targeting phoA and stx1 genes, respectively. Most importantly, no cross relations were observed in 58 bacterial strains making this method a satisfactory monitoring device. Recently detection of SFG *Rickettsia spp.* and *Plasmodium spp.* were also carried out utilizing a loop-mediated isothermal amplification (LAMP) method combined with a dipstick DNA chromatography technology. The targeted genes were detected simultaneously and obtained a sensitivity of 1000 copies per reaction when synthetic nucleotides of *Rickettsia* and *Plasmodium* genes were combined, whereas when native, genomic DNA has been used, a significant drop of sensitivity has been observed. The sensitivity value was 100 and 10 genome equivalents per reaction for *Rickettsia monacensis* and *Plasmodium falciparum*, respectively [73].

As multiplexing is an arduous process and its development requires the meticulous arrangement of multiple primers, a multiplex assay for periodontal pathogen still needs to be developed in order to obtain a more precise and fastidious method, aiding clinicians in their daily practice.

## 4. Materials and Methods

### 4.1. Search Strategy and Data Extraction

This systematic review was conducted up to 30 November 2020, according to the Preferred Reporting Items for Systematic Reviews and Meta-Analyses (PRISMA) statement guidelines [74], using the databases PubMed, Scopus and Web of Science. The search formula included “loop-mediated isothermal amplification” and “periodontal disease” or “periodontal bacteria” or “periodontal pathogen” or “periodontal diagnostics” as terms combined in PubMed Advanced Search Builder. In other databases, analogous combinations of keywords were used.

Records were screened by the title, abstract and full text by two independent investigators. Studies included in this review matched all the predefined criteria according to PICOS (“Population”, “Intervention”, “Comparison”, “Outcomes”, “Study design”)—Table 3. A detailed search flowchart is presented in Figure 1 (in section Results).

The heterogeneity of the detected perio-pathogens, as well as the mainly qualitative character of LAMP results, did not allow us to perform a meta-analysis of the studies included in the present systematic review.

### 4.2. Quality Assessment

The level of evidence was assessed using the classification of the Oxford Center for Evidence-Based Medicine levels for diagnosis [75]. All of the included studies have a low 4th level of evidence (in this 5-graded scale).

## 5. Conclusions

The practicality, rapid use and ease of handling of the LAMP technique through a simple isothermal water device could make it an ideal apparatus for in-office periodontal pathogen screening and monitoring. Advances in LAMP technology, its high sensitivity, and its ability for live monitoring of a reaction might make it a useful tool for proper diagnostic purposes.

## Figures and Tables

**Table 1:** General characteristics of included studies.

Author, Year, Setting	Study Group	Subgingival Plaque Samples	Detected Bacteria
Maeda et al., 2005, Japan [44]	periodontitis patients	by inserting paper points (#45) into periodontal pockets; stored at −20 °C	*Porphyromonas gingivalis*
Yoshida et al., 2005, Japan [45]	10 periodontitis patients	by inserting a sterile endodontic paper point into the subgingival site for 10 s; NR	*Porphyromonas gingivalis, Tannerella forsythia, Treponema denticola*
Osawa et al., 2007, Japan [46]	8 periodontitis patients	by inserting a sterile endodontic paper point into the subgingival site for 10 s; NR	*Aggregatibacter actinomycetemcomitans*
Miyagawa et al., 2008, Japan [47]	periodontitis patients	by inserting paper points (#45) into periodontal pockets; stored at −30 °C	*Aggregatibacter actinomycetemcomitans, Campylobacter rectus, Eikenella corrodens, Fusobacterium nucleatum, Porphyromonas gingivalis, Prevotella intermedia, Treponema denticola, Tannerella forsythia*
Seki et al., 2008, Morocco [48]	adolescent periodontitis patients	on paper points; stored at −20 °C	*Aggregatibacter actinomycetemcomitans*
Elamin et al., 2011, Sudan [49]	17 subjects with localized aggressive periodontitis and 17 subjects with no clinical periodontal attachment loss (controls); any antibiotics for the past 3 months	collected from the deepest periodontal pocket, one sample from each quadrant; by inserting two paper points (#35) into periodontal pockets; stored at −80 °C	*Aggregatibacter actinomycetemcomitans*
Elamin et al., 2017, Sudan [50]	the same as above	the same as above	*Aggregatibacter actinomycetemcomitans, Porphyromonas gingivalis, Tannerella forsythia, Treponema denticola*
Hamzan et al., 2018, Malaysia [51]	clinical signs of periodontitis, presented with periodontal pocket depth equal or exceeding 4 mm with radiographic evidence of alveolar loss; any antibiotics for the past 3 months prior to the sample collection	collected by vertical stroke of curette and transferred on ice	*Porphyromonas gingivalis, Aggregatibacter actinomycetemcomitans*
Su et al., 2019, China [52]	40 patients with periodontitis (20 males and 20 females), ranging from 35 to 55 years of age, selected from those referred for scaling and root planning	collected from the roots of teeth, beyond the gingival margin, using a special brush and delivered to the laboratory on ice; stored at −20 °C	*Porphyromonas gingivalis*

Legend: NR, not reported.

**Table 2:** The parameters of the conducted loop-mediated isothermal amplification (LAMP) analyses.

Study	Reaction Mixture (25-µL Volume)	Reaction Conditions	Detection of Products
Maeda et al., 2004 [44]	40 pmol each FIP and BIP, 5 pmol each F3 and B3c, 1 µL *Bst* DNA polymerase, 2 µL extracted DNA, and 12.5 µL reaction mix; for the acceleration 20 pmol each LFc and LB	incubated at 60, 62, 64 or 66 °C for 30 or 60 min; terminated by heating at 80 °C for 2 min	naked-eye inspection 1.0 µL of 10^−1^ or 10^−3^-diluted SYBR Green I; white turbidity by magnesium pyrophosphate; 2% agarose gel electrophoresis with ethidium bromide staining
Yoshida et al., 2005 [45]	1.6 µM each FIP and BIP, 0.2 µM each F3 and B3, 0.8 µM each LF and LB, 8 U *Bst* DNA polymerase, 1.4 mM each dNTPs, 0.8 M betaine, 20 mM Tris-HCl (pH 8.8), 10 mM KCl, 10 mM (NH_4_)_2_SO_4_, 8 mM MgSO_4_, 0.2% Tween 20, and 5 µL target DNA	incubated at 65 °C; terminated by heating at >80 °C for 2 min	naked-eye inspection 1.0 µL of 10^−1^-diluted SYBR Green I; white turbidity by magnesium pyrophosphate; 2% agarose gel electrophoresis
Osawa et al., 2007 [46]	the same as above	incubated at 67 °C; terminated by heating at >80 °C for 2 min	the same as above
Miyagawa et al., 2008 [47]	40 pmol each FIP and BIP, 5 pmol each F3 and B3, 1 µL *Bst* DNA polymerase (8 U), 2 µL template DNA, and 12.5 µL reaction mixture; for acceleration 20 pmol LB or each LF and LB	incubated at 62, 64 or 66 °C for 60 min; terminated by heating at 80 °C for 2 min	naked-eye inspection 10^−1^-diluted SYBR Green I; 2% agarose gel electrophoresis with ethidium bromide staining
Seki et al., 2008 [48]	1.6 µM each FIP and BIP, 0.2 µM F3 and B3, 0.4 µM LF and LB, 8 U *Bst* DNA polymerase, 1.4 mM each four dNTPs, 0.8 M betaine, 20 mM Tris-HCl (pH 8.8), 10 mM KCl, 10 mM (NH_4_)_2_SO_4_, 8 mM MgSO_4_, 0.1% Tween 20, and template DNA up to 5 µL	incubated at 63 °C for 60 min; terminated by heating at 80 °C for 2 min	white turbidity by magnesium pyrophosphate; 3% agarose gel electrophoresis with ethidium bromide staining
Elamin et al., 2011 [49]	the same as above	the same as above	the same as above
Elamin et al., 2017 [50]	the same as above, except template DNA up to 5.5 μL	incubated at 63, 64 and 65 °C for 60 min; terminated by heating at 80 °C for 2 min	white turbidity by magnesium pyrophosphate; 2% agarose gel electrophoresis with ethidium bromide staining
Hamzan et al., 2018 [51]	1.6 μM each FIP and BIP, 0.2 μM each F3 and B3, 0.4 μM each LF and LB, 320 U/mL *Bst* DNA polymerase, 1.4 mM each dNTPs, 20 mM Tris-HCl (pH 8.8), 150 mM KCl, 10 mM (NH_4_)_2_SO_4_, 8 mM MgSO_4_, 0.1% Tween 20, and 2 μL crude template	incubated at 65 °C for 30 min; terminated by heating at 95 °C for 2 min	naked-eye inspection 1.0 µL of 10^−1^-diluted SYBR Green I; white turbidity by magnesium pyrophosphate; 2% agarose gel electrophoresis with SYBR Safe staining
Su et al., 2019 [52]	40 pmol FIP and BIP, 5 pmol F3 and B3, 20 pmol LF, 8 pmol LB, 8 U *Bst* DNA polymerase, 1.4 mM dNTPs, 0.8 M betaine, 20 mM Tris-HCl (pH 8.8), 10 mM KCl, 10 mM (NH_4_)_2_SO_4_, 8 mM MgSO_4_, 0.1% Tween 20, and 2 μL template DNA	incubated at 65 °C; NR	turbidimeter (MB-LAMP)

Legend: NR, not reported; FIP, forward inner primer; BIP, backward inner primer; LF, loop F; LB, loop B; DNA, deoxyribonucleic acid; dNTPs, deoxy-nucleoside triphosphates.

**Table 3:** Inclusion and exclusion criteria according to PICOS (“Population”, “Intervention”, “Comparison”, “Outcomes”, “Study design”).

Parameter	Inclusion Criteria	Exclusion Criteria
Population	patients with periodontal diseases, aged from 0 to 99 years, both sexes	patients with other oral diseases
Intervention	loop-mediated isothermal amplification method	PCR techniques
Comparison	not applicable	
Outcomes	detected pathogens of marginal periodontium	detected pathogens of periapical periodontium or other plaque bacteria
Study design	case-control, cohort and cross-sectional studies	literature reviews, case reports, expert opinion, letters to editor, conference reports
	published after 2000	not published in English
